# Transcriptome and Weighted Gene Co-Expression Network Analysis to Characterize the Expression of Genes Related to Yield Traits in Yunnan Hulled Wheat

**DOI:** 10.3390/ijms26062542

**Published:** 2025-03-12

**Authors:** Qianchao Wang, Chuanli Zhang, Yirui Guo, Junna Liu, Ping Zhang, Guofei Jiang, Peng Qin

**Affiliations:** College of Agronomy and Biotechnology, Yunnan Agricultural University, Kunming 650201, China2007078@ynau.edu.cn (C.Z.); 2021110026@stu.ynau.edu.cn (J.L.); 2021110031@stu.ynau.edu.cn (P.Z.);

**Keywords:** Yunnan hulled wheat, transcriptome, KEGG, WGCNA

## Abstract

Yunnan hulled wheat (YHW) is a wild ancestor of cultivated wheat and possesses rich genetic variation; however, there is limited research on teff at the molecular level. Therefore, in this study, two lines with large differences in kernel size were used as materials, and their kernels were sampled at 7, 21, 35, and 49 days after flowering; kernel surface area and thousand kernel weight were measured and analyzed; and transcriptome sequencing analysis was also performed, which showed that a total of 88,801 genes were annotated by Kyoto Encyclopedia of Genes and Genomes (KEGG); the functional annotation showed that the key pathways they involved in yield traits were mainly enriched in gycolysis/gluconeogenesis, pentose and glucuronate interconversions, amino sugar and nucleotide sugar metabolism, starch, and sucrose metabolism pathways, of which *TraesCS5B02G356300*, *TraesCS7B02G375300*, *TraesCS7A02G473900*, and *TraesCS2B02G390700* differed significantly in different subgroups; and a significant difference was observed between the two pathways in different subgroups using weighted gene co-expression network analysis (WGCNA) associated with yield traits. Ten core genes were mined from the two modules with the highest correlation with the target traits. These results provide a theoretical reference basis for interpreting the expression patterns of yield trait-responsive genes in YHW, for further conservation and utilization of the germplasm resources of this rare wheat, and for the screening of high-yielding superior varieties.

## 1. Introduction

Yunnan hulled wheat (*T. aestivum* ssp. *yunnanense* King) is a hexaploid wheat germplasm resource endemic to Yunnan Province, China, and is the original cultivated type of common wheat [1,2]. As one of the three subspecies of common wheat endemic to China, YHW is mainly planted in the Lincang, Baoshan, and Simao areas in the lower reaches of the Lancang and Nujiang rivers [3], and it is often planted in forest clearings, turnip and ditch edges, and sloped cultivated mountainous and semi-mountainous areas at an altitude of 1500–2500 m, with an average annual temperature of 15 °C and an annual rainfall of about 1480 mm, and it is uniquely adapted to the special agro-ecosystems of the local area [4]. YHW possesses very excellent agronomic traits and rich genetic variation with excellent characteristics such as barrenness tolerance, drought resistance, germination on spike and stripe rust resistance, which have potential utilization in the research of selecting and breeding new resistant, green, and excellent wheat varieties [5,6].

Changes in external morphology and internal material enrichment are carried out during all periods of seed grain growth and development [7]. The study of the maturation process of crop grain development helps us to understand the changes in the accumulation of substances in the grain as well as gene expression patterns. Currently, some researchers have analyzed the seed quality components of YHW. For example, Zhang et al. [8] have explored the accumulation patterns of some phenolic, amino acid, and sugar substances in YHW, and some scholars have also investigated the resistance of YHW to diseases, such as powdery mildew and leaf blight [9,10]. Thousand kernel weight is one of the most popular agronomic traits in agricultural production and is an important factor in determining yield. Seed size is also crucial for plant evolution, as large seeds are able to accumulate sufficient nutrients for germination and have better tolerance to abiotic stresses, whereas small seeds facilitate the dispersal and reproduction of large numbers of offspring. Miklič V et al. [11] explored the effects of different locations and harvest dates on the yield and thousand-seed weight of different genotypes of sunflower; different doses of nitrogen applied to the thousand-seed weight of kidney bean seeds also had certain effects [12].

Transcriptomics can reveal genome-wide level expression under abiotic stress by sequencing RNA sequences in plants under a particular physiological condition [13]. WGCNA is an algorithm that mines modular information from gene chip expression data and is the tool for identifying the relationship between co-expression modules and traits [14], which clusters genes with similar expression patterns to form modules and associates them with sample traits to mine related core genes [15]. Nowadays, transcriptomics and WGCNA are widely used in plant research: Zhu et al. performed WGCNA of rice transcriptome data under salt stress and identified some new hub genes in rice [16]; Lu et al. [17] compared transcriptomics and weighted gene co-expression correlation network analysis (WGCNA) to reveal the potential regulatory mechanism of carotenoid accumulation in chrysanthemum; Fan et al. [18] identified candidate genes involved in petaloid stamens in peony flowers by transcriptomics and WGCNA. For a long time, a large number of identical or similar backbone parents have been used in wheat breeding, resulting in poor genetic diversity and a narrowing of the genetic base of cultivated wheat, as well as deterioration of the comprehensive traits and lack of wide adaptability of new wheat varieties. However, YHW has the spike fragility (broken spike) and sticky husk (encrustation) characteristic of the wild ancestors of cultivated wheat, such as Urartu wheat, wild dicot wheat, and knapweed, etc., and it is also an important instrumental material for the study of the origin and evolution of modern cultivated hexaploid wheats [2], and the study of the yield traits of YHW has been rarely reported, in view of which, the present study was conducted using YHW Dikemai 1 and Yunmai 0606 as materials, the present study used transcriptomics approach to mine the key metabolic pathways through differential gene expression analysis, combined with the surface area of the grain and thousand grain weight for WGCNA analysis, screening differentially expressed genes, interpreting the expression patterns of genes responsive to yield traits in YHW, and providing a reference basis for the further conservation and utilization of this rare wheat germplasm resource and screening of high-yielding superior varieties.

## 2. Results

### 2.1. Analysis of Yield Indexes of Different Lines of YHW

**Figure 1:**
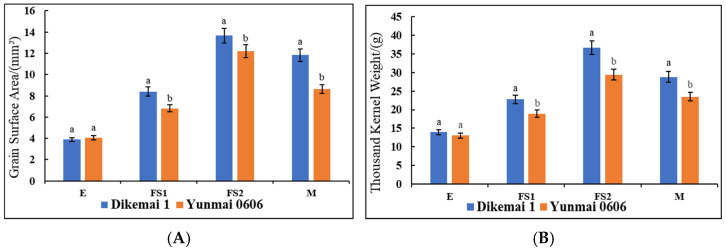
Changes in agronomic traits of YHW at different developmental stages. (**A**) Grain surface area. (**B**) Thousand grain weight. The same letters (a,b) indicate no significant difference (*p* > 0.05), while groups with different letters indicate significant differences (*p* < 0.05).

We determined the changes in the surface area of the kernel as well as the thousand kernel weight of the two types of YHW lines in the periods of E, FS1, FS2, and M (Figure 1A,B). During the E period, there was no significant difference in grain surface area between Yunmai 0606 and Dikemai 1. During the FS1 period, the surface area of Dikemai 1 grains was significantly higher than that of Yunmai 0606. During the FS2 period, both lines showed the fastest increase in grain surface area, reaching the maximum value, and there was a significant difference between the two varieties. In the M stage, the surface area of grains decreased compared to the FS2 stage but was much larger than the FS1 stage. During the four periods, there were also differences in the thousand grain weight between Dikemai 1 and Yunmai 0606. During the E period, there was no significant difference in thousand grain weight between the two varieties. As the grains continue to develop, there have been significant changes in the thousand grain weights of both varieties during the FS1 and FS2 stages. Dikemai 1 has a significantly higher thousand grain weight than Yunmai 0606, but during the FS2 stage, the grains of both varieties are heaviest in all four stages. During the M period, there was a significant difference in the thousand grain weight between the two varieties, but both showed a decrease compared to FS2.

### 2.2. Transcriptome Data Quality Control Analysis

**Figure 2:**
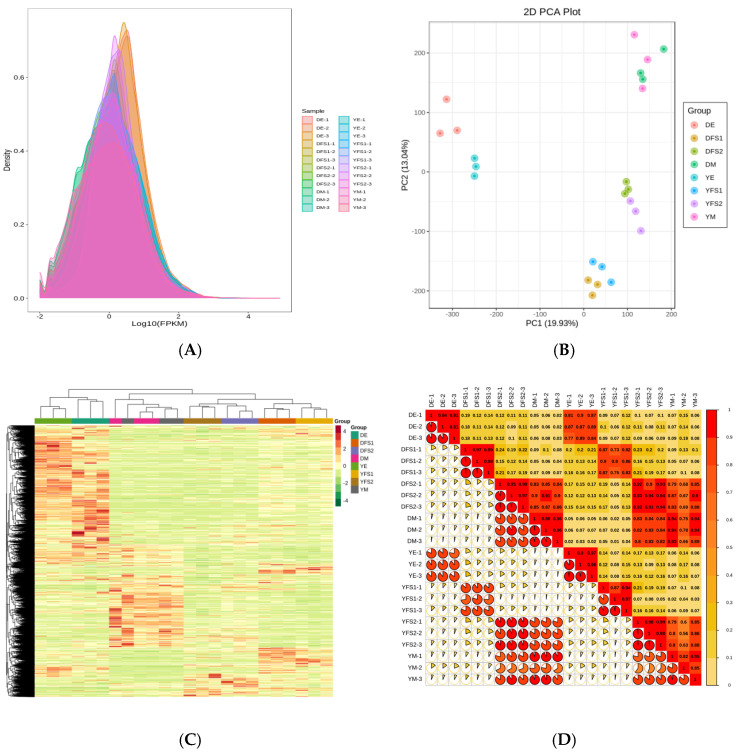
(**A**) Expression density distribution plot. Different colored curves in the graph represent different samples, the horizontal coordinates of the points on the curve indicate the logarithmic values of FPKM of the corresponding samples, and the vertical coordinates of the points indicate the probability densities. (**B**) PCA plot. Each point represents a sample, and samples in the same group are represented using the same color. (**C**) Differential gene clustering heat map. Horizontal coordinates indicate sample names and hierarchical clustering results, vertical coordinates indicate differential genes and hierarchical clustering results. Red color indicates high expression and green color indicates low expression. (**D**) Correlation heat map. Within each circle, the more red parts there are, the stronger the correlation. The squared Pearson correlation coefficient (R^2^) between biological replicate samples should be at least greater than 0.8.

Transcriptome sequencing analysis was performed on 24 samples, and a total of 327.42 Gb of Clean Data were obtained; the Clean Data of each sample reached 10 Gb, with the percentage of Q30 bases at 91% and above. The clean reads after quality control were compared with the reference genome, and the comparison efficiency was found to be high, indicating that the results were reliable and accurate. Expression density distribution graphs were used to show the abundance of genes with changes in expression in different subgroup comparisons, and the FPKM values of gene expression in the experimental samples were concentrated in the range of 10–2.5–102.5 (Figure 2A). The PCA plot shows that there is good repeatability within the sample groups and significant variability between the groups (Figure 2B); combined with the clustering heat map (Figure 2C) and correlation plot (Figure 2D), it can be concluded that there is a difference in gene expression in different subgroups of the comparisons.

### 2.3. Quantitative and Qualitative Gene Expression Analysis

**Figure 3:**
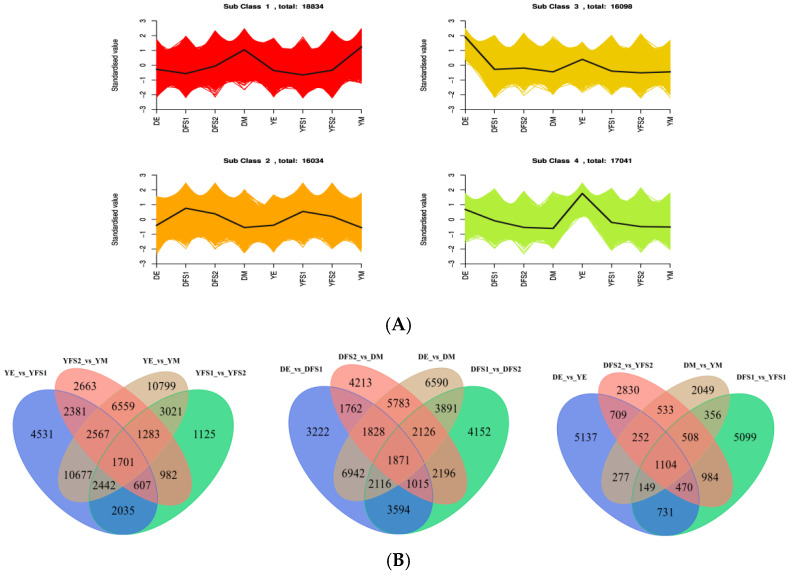
(**A**) K-means plot of DEGs. The x-coordinate represents the sample, and the y-coordinate represents the relative content of standardized genes. The black line represents the overall trend of change. (**B**) Different grouping Venn diagram; each circle represents a comparison group; each circle represents a unique differential gene of the comparison group; and the numbers in the overlapping parts between circles represent the number of common DEGs in the comparison group.

The FPKM of the genes was centered and normalized, and then K-means clustering analysis was performed, which was used to study the expression pattern of the genes. The same class of genes had similar trends under different experimental treatments and may have similar functions (Figure 3A). DEGs were categorized into four clusters (Table S1), with the continuous development of teff, most of the genes showed a gradual upward trend in cluster 1; a downward post-trend in clusters 3 and 4; and an upward and then a downward trend in cluster 2, with the highest at 21 days after anthesis, and these findings can be used as a potential marker to differentiate between genes of teff seed grain growth and development.

After extracting the centered and normalized FPKM expression of DEGs, the hierarchical clustering analysis was performed and the clustering heatmap of each differential grouping was plotted (Figure S1), which shows that the results of hierarchical clustering of differential gene expression in different groupings are different. The Venn diagram (Figure 3B) analysis showed a total of 1104 DEGs in DE vs. YE, DFS1 vs. YFS1, DFS2 vs. YFS, and DM vs. YM, and 5137, 2830, 5099, and 2049 DEGs in each of them (Figure 3B, Table S2). In Dikemai 1, there were a total of 1871 DEGs in DE vs. DFS1, DFS1 vs. DFS2, DFS2 vs. DM, and DE vs. DM with 3222, 4152, 4213, and 6590 respective DEGs as the seeds continued to develop (Figure 3B, Table S3). In Yunmai 0606, there were a total of 1701 DEGs in YE vs. YFS1, YFS1 vs. YFS2, YFS2 vs. YM, and YE vs. YM, with 4531, 2663, 10799, and 1125 respective DEGs as the seeds continued to develop (Figure 3B, Table S4).

### 2.4. Functional Annotation Analysis of DEGs

Functional annotation of the detected 147,201 genes in the NR, Swiss-Prot, GO, Tremble, KOG, Pfam, and KEGG databases revealed that 138,970 genes were annotated in NR; 84,093 genes were annotated in Swiss-Prot; 27,137 genes were annotated in GO; and 103,549 genes were annotated in KOG; Pfam function annotated to 100,153 genes; KEGG function annotated to 88,801 genes and 144,061 genes in Trembl. DEGs between sample groups were performed using DESeq2 to obtain sets of DEGs between two biological conditions, with DEGs screened for |log_2_Fold Change| ≥ 1 and FDR < 0.05. Statistics of the total number of DEGs, the number of up-regulated genes, and the number of down-regulated genes in each group are shown in Table 1.

**Figure 4:**
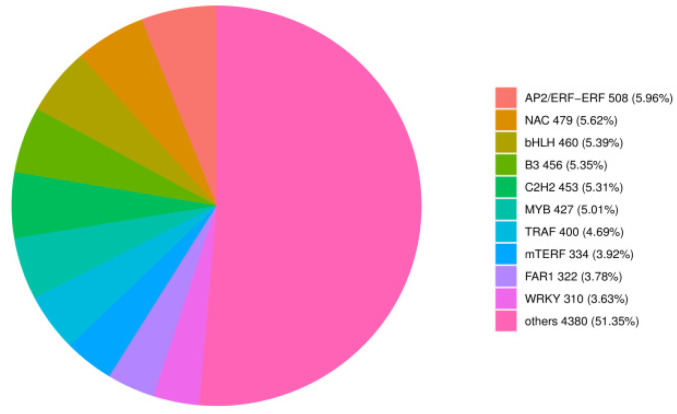
Transcription factor classification diagram. Different colors indicate different families.

Further investigating the transcripts of TF-encoding genes, we identified 8529 TF-encoding genes from 90 different families (Table S5). The top nine TFs were AP2/ERF-ERF, NAC, bHLH, B3, C2H2, MYB, TRAF, mTERF, and WRKY families (Figure 4).

### 2.5. Enrichment Analysis of Differentially Expressed Genes

**Figure 5:**
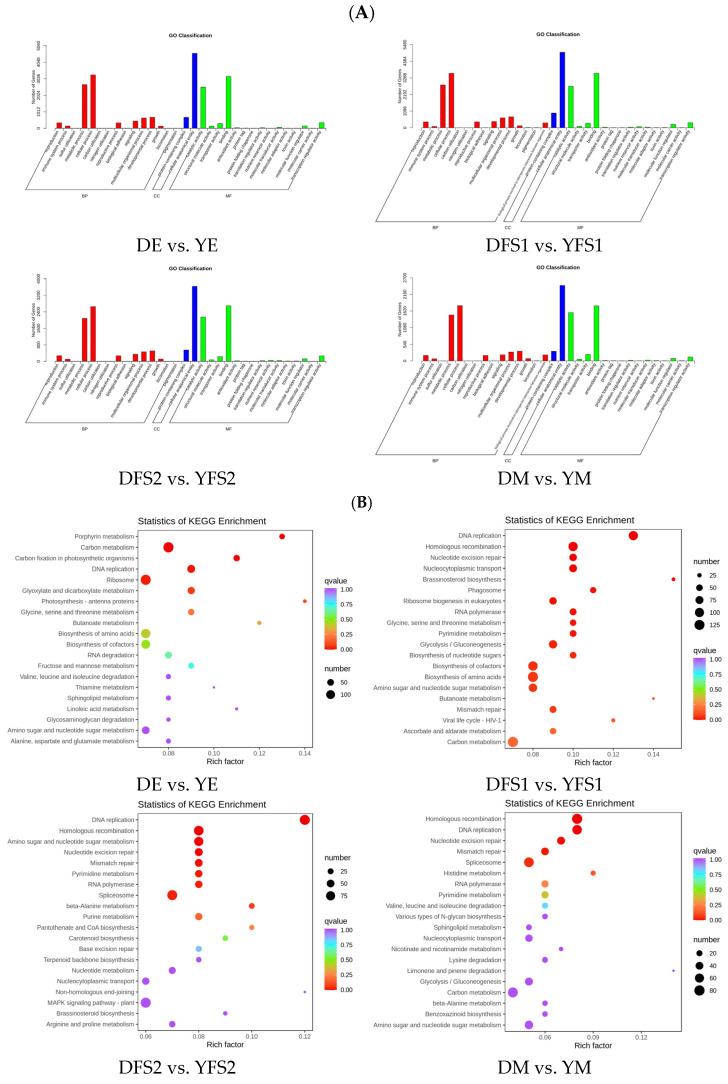
(**A**) Bar chart of GO classification. Horizontal coordinates indicate secondary GO entries and vertical coordinates indicate the number of differential genes in GO entries. (**B**) Enrichment scatter plot. Vertical coordinates indicate KEGG pathways; horizontal coordinates indicate the Rich factor; the larger the Rich factor is, the greater the degree of enrichment is; vertical coordinates indicate KEGG pathways; horizontal coordinates indicate Rich factor. The larger the rich factor, the greater the degree of enrichment; the larger the point, the greater the number of DEGs enriched in the pathway; the redder the color of the point, the more significant the enrichment.

To further analyze the DEGs, we annotated them to GO and KEGG enrichment analyses. The GO clustering analysis of DEGs showed that the DEGs in the comparison group were mainly concentrated in three major categories: biological process (BP), molecular function (MF), and cellular component (CC) (Figure 5A).

The results of KEGG enrichment analysis are presented in the form of a scatterplot, in which the degree of KEGG enrichment is determined based on the Rich factor, Q-value, and the number of genes in the given pathway (Figure 5B). The Rich factor is the ratio of the number of DEGs in the given pathway to the total number of annotated genes. The larger the Rich factor, the greater the enrichment. The smaller the Q value, the more significant the enrichment. For each comparison, the 20 pathway entries that were most significantly enriched; if fewer than 20 pathway entries were enriched, all are shown. DEGs were found to be mainly enriched in metabolic pathways, biosynthesis of secondary metabolites, glycolysis/gluconeogenesis, pentose and glucuronate interconversions, amino sugar and nucleotide sugar metabolism, starch, and sucrose metabolism, carbon metabolism, carbon fixation in photosynthetic organisms pathways (Figure 5B).

### 2.6. Modeling of Candidate Gene Network Regulation in Response to Important Yield Traits

**Figure 6:**
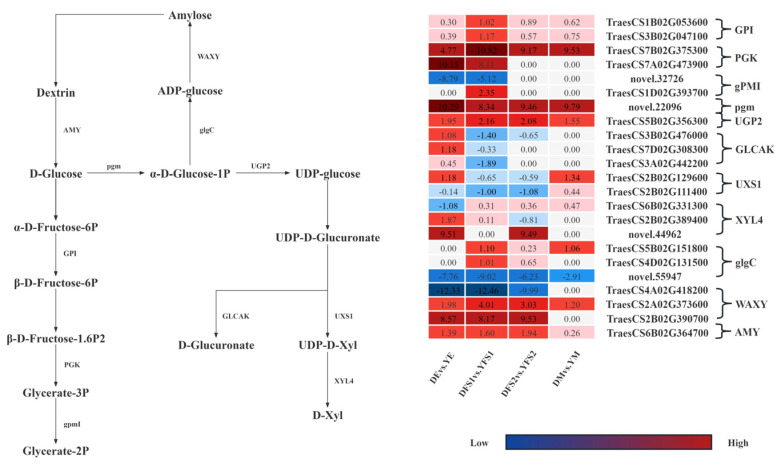
Diagram of DEGs response mechanisms at different developmental periods of iron hulled wheat grains. The gene expression levels are expressed as FPKM values. The red color indicates a positive value, the blue color indicates a negative value, and the darker the color, the larger the value.

In order to further compare the gene expression of the two iron hulled wheats at the same developmental period, we analyzed the gene expression in the glycolysis/gluconeogenesis, pentose and glucuronate interconversions, Amino sugar and nucleotide sugar metabolism, starch, and sucrose metabolism pathways were analyzed, and some differential genes were screened out and gene expression network maps were drawn (Figure 6).

In the glycolysis/gluconeogenesis pathway, most of the structural genes related to glucose-6-phosphate isomerase (GPI), phosphoglycerate kinase (PGK), 2,3-bisphosphoglycerate-independent phosphoglycerate mutase (gPMI), and phosphoglucomutase (pgm) had significantly different expression levels in DE vs. YE, DFS1 vs. YFS1, DFS2 vs. YFS2, and DM vs. YM, where genes *TraesCS1B02G053600*, *TraesCS7B02G375300*, *TraesCS7A02G473900*, and *novel.22096* were significantly up-regulated and expressed in all four subgroups; *novel.32726* was significantly down-regulated and expressed in DE vs. YE, DFS1 vs. YFS1.

In the pentose and glucuronate interconversions pathway, the UTP-glucose-1-phosphate uridylyltransferase (UGP2) related structural gene *TraesCS5B02G356300* was significantly up-regulated in DE vs. YE, DFS1 vs. YFS1, DFS2 vs. YFS2, and DM vs. YM. The related structural genes in the pentose and glucuronate interconversions (GLCAK). The genes *TraesCS3B02G476000*, *TraesCS7D02G308300*, and *TraesCS3A02G442200* were only up-regulated for expression in DE vs. YE, down-regulated for expression in DFS1 vs. YFS1, and insignificantly up-regulated for expression in DFS2 vs. YFS2, DM vs. YM. Similarly, in the amino sugar and nucleotide sugar metabolism pathway, the structural gene *TraesCS2B02G129600*, which is related to UDP-glucuronate decarboxylase (UXS1), was up-regulated for expression in DEvs.YE, DMvs.YM, and down-regulated for expression in DFS1 vs. YFS1, DFS2 vs. YFS2; *TraesCS2B02G111400* was down-regulated for expression in DE vs. YE, DFS1 vs. YFS1, DFS2 vs. YFS2 and up-regulated for expression in DM vs. YM.

In the starch and sucrose metabolism pathway, the structural genes *TraesCS5B02G151800* and *TraesCS4D02G131500*, which are related to glucose-1-phosphate adenylyltransferase (glgC), were up-regulated and expressed in DFS1 vs. YFS1, DFS2 vs. YFS2; whereas the gene novel.55947 was significantly down-regulated in DE vs. YE, DFS1 vs. YFS1, DFS2 vs. YFS2, and DM vs. YM were significantly down-regulated for expression. Granule-bound starch synthase (WAXY) and alpha-amylase (AMY) related structural genes *TraesCS2A02G373600*, *TraesCS2B02G390700*, and *TraesCS6B02G364700* were significantly up-regulated and expressed in DE vs. YE, DFS1 vs. YFS1, DFS2 vs. YFS2; WAXY related structural genes *TraesCS4A02G418200* were significantly down-regulated in DE vs. YE, DFS1 vs. YFS1, DFS2 vs. YFS2.

### 2.7. Screening of Important Candidate Genes for Grain Yield Traits in Teff Wheat Using WGCNA

**Figure 7:**
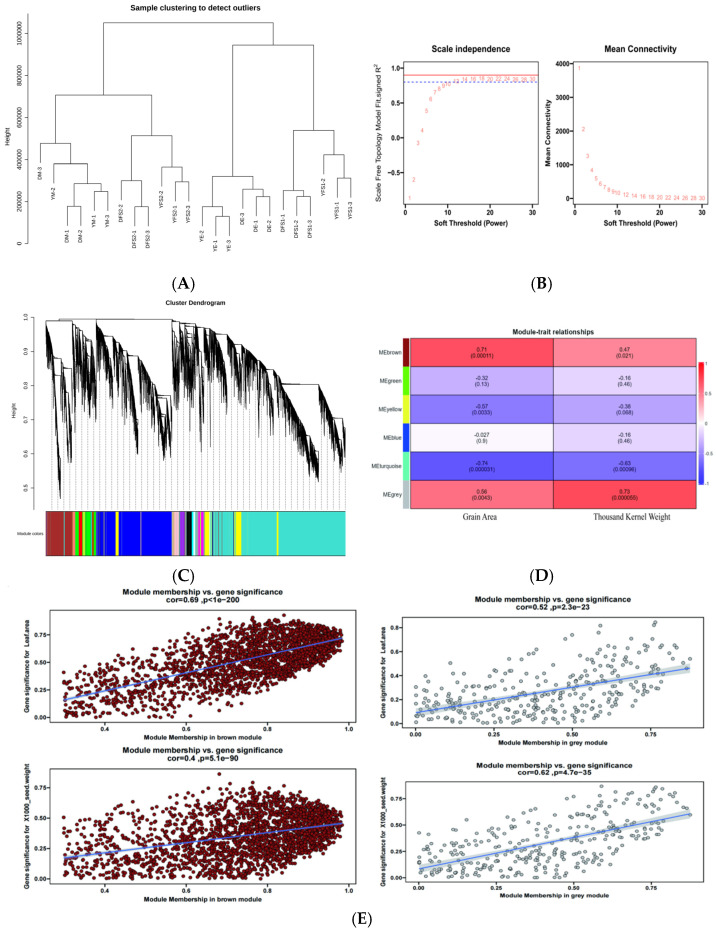

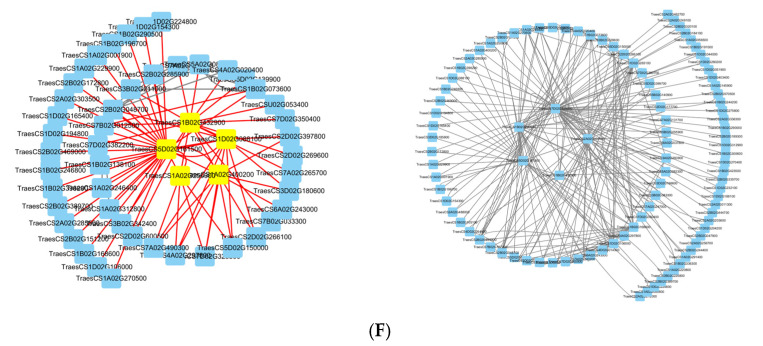
(**A**) Sample clustering diagram of iron hulled wheat seeds. Horizontal coordinates represent sample clustering, one column represents one sample, and clustering is based on the similarity of gene expression between samples, the closer the gene expression between samples, the closer they are to each other. (**B**) Network topology with different soft-threshold powers; *x*-axis denotes the weight parameter β; *y*-axis of the left plot denotes the squared correlation coefficients between log(k) and log(p(k)) in the corresponding network. The *y*-axis of the right graph represents the average of the neighbor-joining functions of all genes in the corresponding gene module. Approximate scale-free topology is obtained at a soft threshold power of 16 for both genotypes. (**C**) Gene modules identified by WGCNA. Gene tree plots were obtained by clustering the corresponding modules differently based on consistent topological overlap and color clustering of the corresponding modules indicated by color rows. Each row represents a color-coded module containing a set of highly connected genes. (**D**) Heat map of the association of gene co-expression network modules with surface area and thousand kernel weight of iron hulled wheat kernels. Each row corresponds to a consensus module and each column to a time point. Module names are shown on the *y*-axis and time points are shown on the *x*-axis. The table is color-coded by correlation according to the color legend. The strength and direction of the correlations are shown on the right side of the heatmap (red, positive correlation; blue, negative correlation). (**E**) Scatter plots of gene significance (GS) versus module membership (MM) in the MEbromn and MEgrey modules. There is a significant correlation between GS and MM in both modules. (**F**) Plot of candidate hub genes in the MEbromn and MEgrey modules obtained from interaction network analysis with known core genes. The five middle genes are the screened core genes.

After filtering the low-expression genes, a total of 10,000 genes were used to construct a weighted gene co-expression network. Cluster analysis was performed based on the expression of the genes to test whether there were outlier samples, and the results showed that there were no outlier samples (Figure 7A). After that, the pickSoftThreshold function was used to calculate and select the appropriate weighting coefficient β. The selection criteria of β is to satisfy the correlation coefficient squared close to 0.85, and also to ensure a certain degree of gene connectivity. β value was taken as 15 to construct the co-expression network (Figure 7B). Subsequently, the dynamic cutting method was used to divide the network modules, and the small modules with high similarity were merged; finally, twenty-five modules were constructed, with different colors indicating different modules, and the genes in the modules might be highly correlated with the yield traits of hulled wheat (Figure 7C).

For each module, gene co-expression is summarized by feature genes (the expression of the first component of genes belonging to that module), and we calculated the correlation between each feature gene and sample processing conditions, such as grain surface area and thousand grain weight. Based on the correlation between modules and sample processing, using cor > 0.5 and *p* < 0.05 as standards, two gene co-expression modules specifically related to yield traits were identified in this experimental study. The MEgrey module showed a positive correlation with the surface area (r = 0.56, *p* = 0.0043) and thousand grain weight (r = 0.73, *p* = 0.000055) of ironshell wheat grains (Figure 7D). The MEbroman module also showed a positive correlation with the surface area (r = 0.71, *p* = 0.00011) and thousand grain weight (r = 0.47, *p* = 0.021) of ironshell wheat grains (Figure 7D), and the genes enriched in different modules were different (Figure S2); Subsequently, these two modules will be further studied as specific modules related to yield traits of ironclad wheat, and core genes in the modules will be identified.

Considering that the MEbromn and MEgrey blocks have a relatively high correlation with the yield traits of teff and may have genes related to potential response to the yield traits of teff, these 2 blocks were utilized to construct a gene interactions network. Scatter plots of GS and MM values of MEbromn and MEgrey blocks were plotted (Figure 7E). Key genes were screened by setting GS to >0.4 and |MM| > 0.8. We obtained 1349 (Table S6), and 532 hub genes (Table S7) in MEgrey and MEbromn blocks, respectively.

The genes with KME values in the top twenty were selected as initial candidate genes. Subsequently, the BC (Betweenness) values of the candidate genes were calculated by the cytoNCA plug-in in cytoscape 3.9.1 software to screen the core genes, and the five core genes of each module were finally identified. The blue module core genes are *TraesCS5D02G161500*, *TraesCS1B02G432900*, *TraesCS1D02G088100*, *TraesCS1A02G400200*, and *TraesCS1A02G250900*; the dark gray module core genes are *TraesCS7D02G325500*, *TraesCS5D02G161500*, *TraesCS2A02G303500*, *TraesCS1B02G432900*, and *TraesCS1B02G290500* (Figure 7F).

### 2.8. Screening of Important Candidate Genes for Grain Yield Traits in Teff Wheat Using RT-qPCR

**Figure 8:**
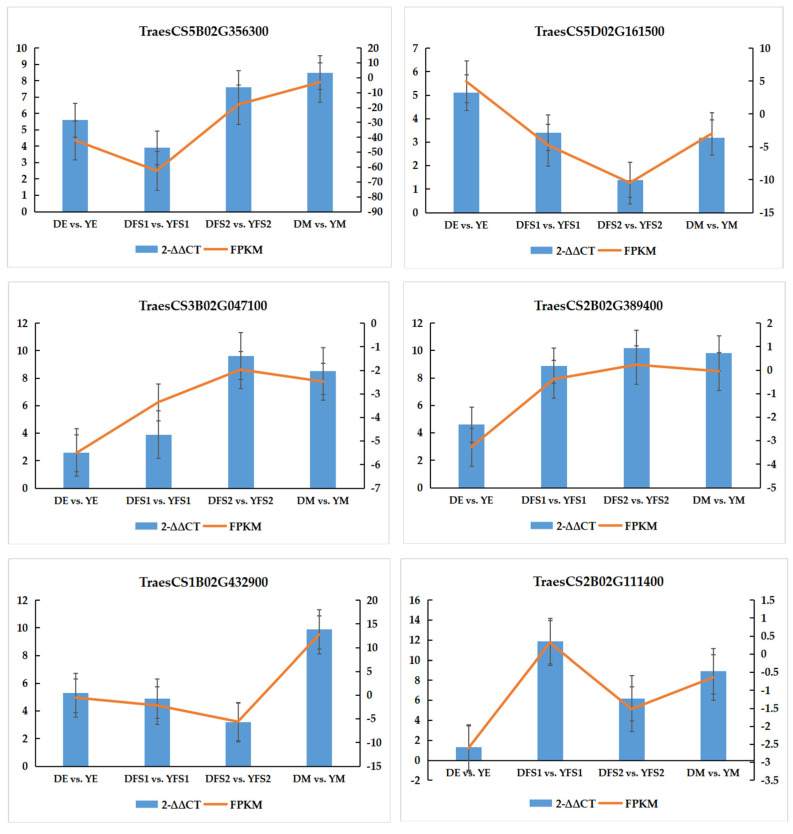
RT-qPCR validation plot.

In order to verify the authenticity and reliability of the transcriptome data and the differential expression level of the candidate genes, real-time fluorescence quantitative PCR was performed on randomly selected differentially expressed genes, and the results showed that the RT-qPCR results of the validated genes were consistent with the RNA-seq data, indicating that the transcriptome sequencing results were reliable (Figure 8).

## 3. Discussion

Seeds are the source of the seed industry and the source of food production. Wheat, rice, and maize are known as the world’s three major food crops [19], of which wheat, in turn, as one of the most important staple foods for human beings, contributes very much to the total global food production, and its importance in global food production cannot be ignored.

As a kind of germplasm resource with perfect retention of original traits, the important value of YHW should not be neglected. The study took two YHW lines with significant seed size differences as materials, and we carefully measured the thousand kernel weights and seed surface areas of these two varieties at 7, 21, 35, and 49 days after anthesis, respectively. We analyzed the key genes affecting the grain sizes and yields of YHW in the process of grain development and found that there were genes with promoting effects on the yield. We also analyzed the key genes affecting seed size and yield during the seed development of YHW, found the genes that have a promotional effect on yield and explored in depth the mechanism of regulating seed size of Tetrahymena, which will lay a theoretical foundation for the utilization of this subspecies in yield breeding of wheat in Yunnan Province.

It has been shown that the development of wheat caryopsis can be roughly divided into three stages. In the first stage (0–14 days after anthesis), division and expansion take place, the basic structure of the grain is established, and grouting begins; in the second stage of “grain grouting” (grain begins to deposit starch granules, 14–28 days after anthesis), the dry weight of the grain increases about twice; after 28 days after anthesis, the rate of grouting of the grain slows down and is completed in about 35 days, and the fresh weight decreases rapidly after 42 days due to drying [20]. This is in general agreement with the results of our study on YHW, in which the thousand kernel weight of YHW at 49 days after anthesis was also lower than that at 35 days after anthesis. The process of crop grain growth and development can be generally categorized into the irrigated, milk-ripe, wax-ripe, and finish-ripe stages [21]. In order to investigate the changes in gene expression during these stages, especially their effects on seed size regulation, we sampled two YHW lines with large differences in kernel size at 7, 21, 35, and 49 days after anthesis and identified these key nodes for transcriptomics analysis. PCA and differential expression analyses revealed significant differences in gene expression profiles between the two varieties at different periods, with the FS period representing the critical period for significant seed size differences between the two materials, suggesting that changes in gene expression at the onset of the indicated irrigational period may have a decisive impact on the outcome of seed size in YHW.

Seed development can be divided into endosperm development, cell division, embryo and cotyledon differentiation, embryo development, seed dehydration, and carbohydrate accumulation [22]. Generally, cell differentiation in wheat seeds is completed 14–28 days after flowering. At this stage, the cells have greatly proliferated and seed size is increasing while proteins and carbohydrates are continuously produced in the seed [23]. The regulation of seed size involves multiple signaling pathways including photosynthesis, carbon metabolism, and hormone signaling pathways [24], which regulate seed size by controlling the development of embryo and endosperm as well as proliferation and growth of seed coat or hull cells [25]. In our study, 14–35 days after anthesis was the critical period for size enlargement and weight gain of YHW seeds, and the results of seed weight analysis during development showed that 21 and 35 days after anthesis were the critical periods for seed matter accumulation/weight gain of YHW. KEGG pathway analysis showed that metabolic pathways, biosynthesis of secondary metabolites, glycolysis/gluconeogenesis, pentose and glucuronate interconversions, amino sugar and nucleotide sugar metabolism, starch and sucrose metabolism, carbon metabolism, and carbon fixation in photosynthetic organisms were significantly enriched. Glycolysis plays a very important role in carbon metabolism and plant development by converting sucrose produced by photosynthesis into precursors for protein and fatty acid biosynthesis [26,27]; therefore, these pathways are closely related to seed formation, which is in agreement with our seed weight findings.

We used differential expression, clustering, and WGCNA analyses to screen some candidate genes from the transcriptome data that might affect seed yield and validated them by qRT-PCR. During grain development, both sugar and starch metabolism are active during grain development [28]. The grain-filling stage is a critical period for starch synthesis in the endosperm. In this study, the structural genes *TraesCS1B02G053600* and *TraesCS3B02G047100* related to GPI in Dikemai 1 were significantly higher than Yunmai 0606 in the glycolysis/gluconeogenesis pathway at all four stages. GPI as a key enzyme in starch synthesis and metabolism pathways, is an important substance connecting the Calvin cycle, starch synthesis metabolism, and sugar metabolism. It can catalyze the mutual transformation of 6-phosphate glucose and 6-phosphate fructose, which are important intermediates in the Calvin cycle, starch synthesis metabolism, and sugar metabolism, and play an important role in grain quality and yield.

PGK and gpmI are both key catalytic enzymes in the glycolysis pathway. The structural genes *TraesCS7B02G375300* and *TraesCS7A02G473900* related to PGK were significantly upregulated. PGK catalyzes the production of 3-phosphoglycerate and ATP from 1,3-diphosphoglycerate and ADP in the glycolysis pathway, ensuring energy supply during starch synthesis during the grain-filling stage [29]; pgm is involved in sucrose synthesis, and its related structural gene *novel.22096* is present in DE vs. YE, DFS1 vs. YFS1, DFS2 vs. YFS2, and DM vs. YM were significantly upregulated, which may promote the synthesis of sucrose, a key transport form of photosynthetic products, ultimately increasing seed yield [30]. In the starch and sucrose metabolism pathway, the structural genes *TraesCS5B02G151800* and *TraesCS4D02G131500* associated with glgC are involved in DFS1 vs. YFS1, DFS2 vs. YFS2 upregulated expression, *Novel. 55947* was downregulated in all four periods. WAXY, as a key enzyme in the synthesis of amylose, mainly controls the synthesis of amylose in storage organs such as seeds, embryos, and endosperm, and is stored in endosperm cells [31]. The structural gene *TraesCS4A02G418200* related to WAXY is expressed in DE vs. YE, DFS1 vs. YFS1, DFS2 vs. YFS2 downregulated expression may be the gene-specific expression of Yunmai 0606 at different reproductive stages, while *TraesCS2B02G390700* and *TraesCS2A02G373600* were downregulated in DE vs. YE, DFS1 vs. YFS1, DFS2 vs. YFS2, and DM vs. YM are all upregulated in expression. At the same time, WAXY also has the function of starch decomposition to generate ADP, which is related to energy metabolism in the glycolysis/gluconeogenesis pathway. The structural gene *TraesCS6B02G364700* associated with AMY is expressed in DE vs. YE, DFS1 vs. YFS1, DFS2vs. YFS2, and DM vs. YM are all upregulated in expression. AMY can regulate starch synthesis and assist in energy supply, and enhance the stress resistance of grains during maturation and dehydration, maintaining the physiological activity and growth and development of YHW grains.

GPI, PGK, gpmI, pgm, glgC, WAXY, and AMY are key enzymes regulating endosperm starch accumulation in ironclad wheat. Their expression levels can affect the synthesis and accumulation of starch in grains, and the expression of these starch-related synthase genes at different stages of grain development jointly affects the final weight of ironclad wheat. Research has shown that wheat with high starch content has higher expression levels of starch synthase genes than wheat with low starch content [32], this is consistent with our research findings.

UGP2 is mainly involved in the process of sugar metabolism. Its main function is to convert α-D-Glucose-1-phosphate into UDP-glucose, which plays an important role in glycogen synthesis and sugar metabolism pathways [33]. Among them, the structural gene *TraesCS5B02G356300* related to UGP2 is present in DE vs.YE, DFS1 vs. YFS1, DFS2 vs. YFS2, and DM vs. YM, and was significantly upregulated in expression. GLCAK is also significantly expressed in the pentose and glucuronate interconversion pathway. It not only participates in metabolic processes such as starch and glucose but also regulates starch synthesis and quality, thereby affecting the quality and yield of YHW grains. For example, GLCAK in wheat grains can regulate starch synthesis and quality, thereby affecting the overall quality and yield of wheat [34,35].

The amino sugar and nucleotide sugar metabolism pathway is connected to the pentose and glucuronate interconversion pathway through the D-Glucuronate. In this study, the activity of UXS1 in amino sugar and nucleotide sugar metabolism significantly changes. As an important enzyme in sugar metabolism, UXS1 can catalyze the conversion of UDP glucuronic acid to D-Xyl. In this study, the XYL4 structural gene related to UDP xylose was identified in DE vs. YE, DFS1 vs. YFS1, and DFS2 vs. YFS2 is significantly expressed, and UDP xylose can also act as a glycosylation modification for cell wall synthesis and storage substances in seeds, providing evidence for the most critical developmental period of iron shell wheat grains, which is the grain filling stage. Some key enzymes in the amino sugar and nucleotide sugar metabolism pathway degrade nucleic acids to bases, pentose, and phosphate. Pentose, in turn, is involved in sugar metabolism. Therefore, the expression of genes in the amino sugar and nucleotide sugar metabolism pathway participates in the complex metabolic network of organisms, which provides an important material basis for the physiological status and function of YHW, the growth, and development of the kernel, and the metabolism to increase yield.

## 4. Materials and Methods

### 4.1. Plant Material

**Figure 9:**
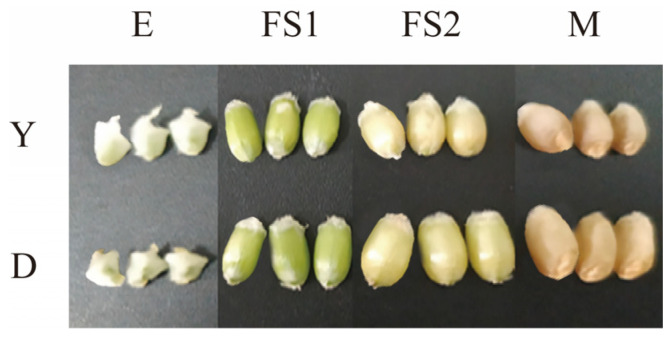
Grain maps of Dikemai 1 and Yunmai 0606 at different developmental stages.

Using Dikemai 1 (from Lincang Agricultural Science Research Institute, Lincang, China, referred to as D, Lincang hulled wheat, slightly white) and Yunmai 0606 (from Yunnan province crop germplasm resources preservation bank, Kunming, China, referred to as Y, Lancang hulled wheat, slightly red skin) as materials (Figure 9), they were planted in the Modern Agricultural Education and Research Base of Yunnan Agricultural University in Xundian County, Kunming (25°24′ N, 102°43′ E). Each line was collected at four different growth stages 7, 21, 35, and 49 days after flowering, labeled as: DE\YE (grain enlargement stage, 7DAF), DFS1\YFS1 (grain filling stage 1, 21DAF), DFS2\YFS2(grain filling stage 2, 35DAF), and DM\YM (grain mature stage, 49DAF) (Table 2). Three biological replicate samples were collected at each stage of grain development in two strains, each containing 10 wheat spikes of the same size. After sampling, 18 samples were rapidly frozen in liquid nitrogen and stored at −80 °C for transcriptome analysis.

### 4.2. Transcriptome Sequencing and Data Analysis

RNA extraction, RNA detection, library construction, on-board sequencing, and bioinformatics were performed and analyzed at Wuhan Metwel Biotechnology Co., Ltd. (Wuhan, China, www.metware.cn, accessed on 30 October 2024). After library construction, preliminary quantification was performed with a Qubit 2.0 Fluorometer, followed by insert size detection of the library using an Agilent 2100 bioanalyzer (Agilent Technologies, Santa Clara, CA, USA) and accurate quantification by qRT-PCR. The libraries were then used for online sequencing on the Illumina HiSeq platform. The data were filtered to obtain Clean Data and compared with the reference genome. Genes were annotated using HISATv2.1.0 software [36] and the bowtie2 method [37], and the new genes were compared with KEGG, GO, NR, Swiss-Prot, trEMBL, and KOG databases using BLAST software (2.2.26). FPKM was used as an indicator of gene expression level. Differential expression analysis between sample groups was first performed by DESeq2, and then the hypothesis testing probability (*p*-value) was corrected for multiple hypothesis testing using the Benjamani–Hochberg method to obtain the False Discovery Rate (FDR), and the final differential gene was screened with the conditions |log_2_Fold Change| ≥ 1 and FDR < 0.05.

### 4.3. RT-qPCR

RNA extracted from wheat kernels of different developmental periods was used for RT-qPCR. Each reaction was repeated three times, primers were designed using BeaconDesign 7.9, the internal reference gene was ATP-dependent 26S proteasomal regulatory subunit (26S), and the reagents were PerfectStartTM SYBR qPCR Supermix (TransGen Biotech, Beijing, China), and a StepOnePlus instrument (Applied Biosystems, Foster City, CA, USA) was used to take 2^−ΔΔCt^ to analyze the normalized expression of each sample [38].

## 5. Conclusions

We analyzed seed surface area and thousand grain weight, transcriptome, and WGCNA of teff wheat at 7, 21, 35, and 49 days after anthesis. We found significant differences in seed surface area and thousand grain weight among varieties during seed development; through the transcriptome, we proposed that gycolysis/gluconeogenesis, pentose and glucuronate interconversions, amino sugar and nucleotide sugar metabolism, and starch and sucrose metabolism pathways to explain the response mechanisms associated with yield traits in steelhead wheat; 10 core genes were screened by WGCNA. These genes are the key factors leading to different yields in YHW, and the findings of this study will help breeders screen excellent YHW varieties with high yields, and further conserve and utilize this rare wheat germplasm resource.

## Figures and Tables

**Table 1:** Statistical table of DEGs in different groups.

Compared Samples	Total Number of DEGs with Significant Difference	Total Number of DEGs Significantly Up-Regulated	Total Number of DEGs Significantly Down-Regulated
DE vs. YE	8829	4807	4022
DFS1 vs. YFS1	9401	4540	4861
DFS2 vs. YFS2	7390	3322	4068
DM vs. YM	5228	2985	2243
DE vs. DFS1	22,350	10,450	11,900
DFS1 vs. DFS2	20,961	10,758	10,203
DFS2 vs. DM	20,794	11,539	9255
DE vs. DM	31,147	17,143	14,004
YE vs. YFS1	26,941	13,849	13,092
YFS1 vs. YFS2	13,196	7599	5597
YFS2 vs. YM	18,743	10,370	8373
YE vs. YM	39,049	22,278	16,771

**Table 2:** Corresponding number–name of the different periods of YHW.

Material Name	Days After Anthesis	Number–Name
Dikemai 1	7	DE
	21	DFS1
	35	DFS2
	49	DM
Yunmai 0606	7	YE
	21	YFS1
	35	YFS2
	49	YM

## Data Availability

The original contributions presented in this study are included in the Supplementary Materials. Further inquiries can be directed to the corresponding author.

## Supplementary Materials

The supporting information can be downloaded at https://www.mdpi.com/article/10.3390/ijms26062542/s1.
