# Supplementary figures and images for: Transcriptome and Weighted Gene Co-Expression Network Analysis to Characterize the Expression of Genes Related to Yield Traits in Yunnan Hulled Wheat
Source: Int J Mol Sci. 2025 Mar 12;26(6):2542. doi: 10.3390/ijms26062542 (PMC11942552; doi:10.3390/ijms26062542)

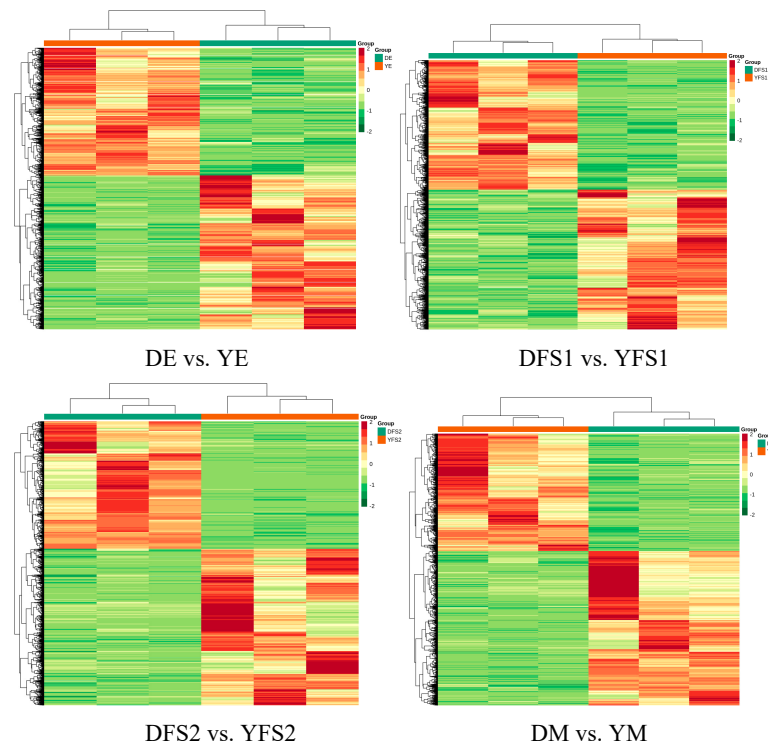

Fig.S1. Heatmap of differential genes clustering for different subgroups

Supplement: Supplementary file 1 [file ijms-26-02542-s001.zip › Fig.S1.pdf]

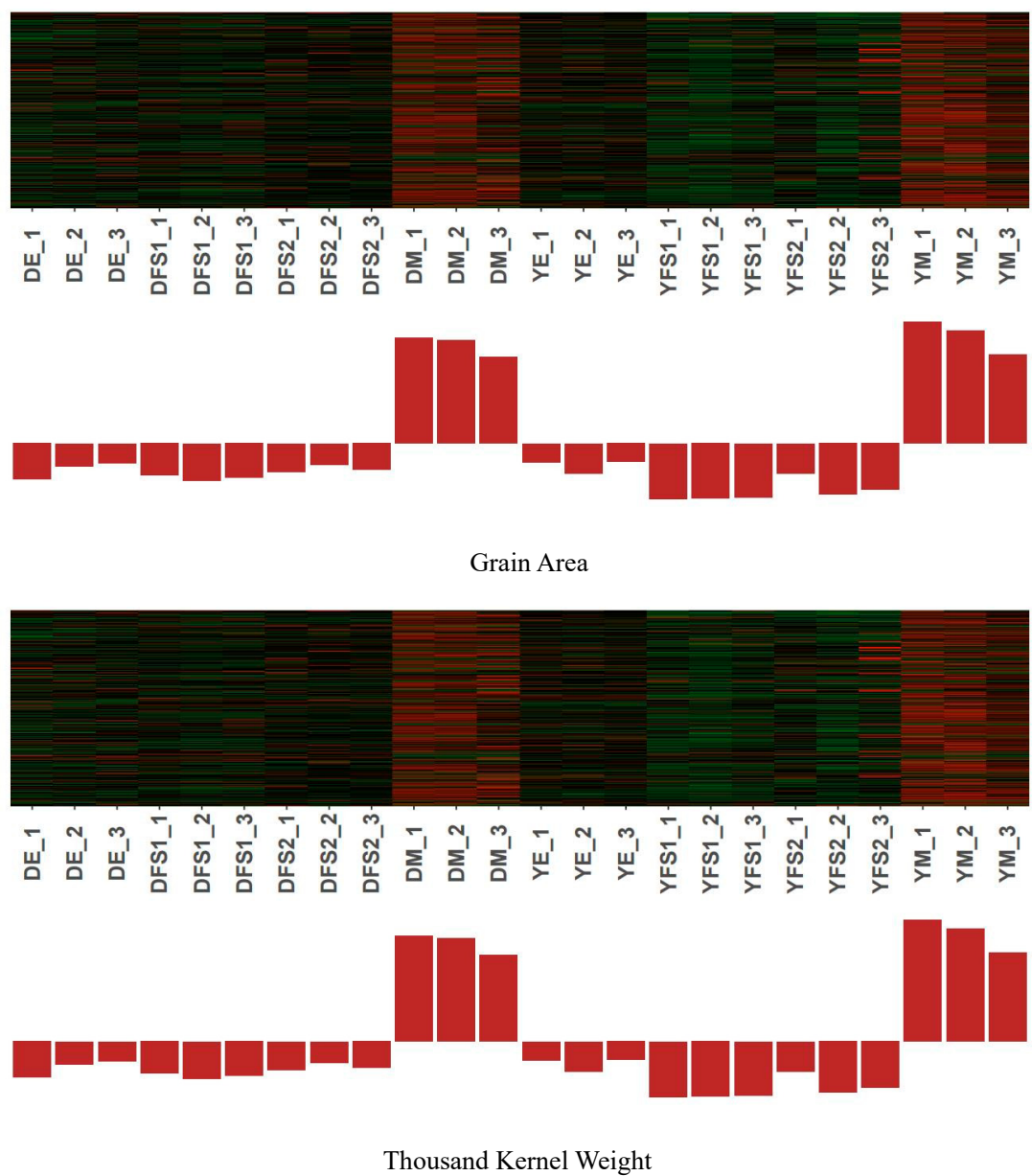

Fig.S2. Heatmap of genes enriched in different modules

Supplement: Supplementary file 1 [file ijms-26-02542-s001.zip › Fig.S2.pdf]
